# The effects of headgear treatment timing on costs and orthodontic outcome: a follow-up study

**DOI:** 10.2340/aos.v85.46468

**Published:** 2026-07-16

**Authors:** Matti Hannula, Mimmi Tolvanen, Pertti Pirttiniemi, Kirsi Pirilä-Parkkinen, Anna-Sofia Silvola, Johanna Julku

**Affiliations:** a Research Unit of Population Health, Faculty of Medicine, University of Oulu, Oulu, Finland; b Medical Research Center, Oulu University Hospital and University of Oulu, Oulu, Finland; c National Rescue Services College, Kuopio, Finland; d Department of Oral and Maxillofacial Diseases, Oulu University Hospital, Oulu, Finland; e Faculty of Medicine and Health Technology, Tampere University, Tampere, Finland; f Pirkanmaa Wellbeing Services County, Tampere, Finland

**Keywords:** Cervical headgear, costs, outcome

## Abstract

**Objective:**

To analyze the effects of cervical headgear (CHG) timing on costs and orthodontic outcome.

**Material and methods:**

Sixty-seven participants with Class II malocclusion were randomized into two groups. In the early group (EG, *n* = 33), CHG treatment began after the eruption of first upper molars. In the later-timed group (LG, *n* = 34), CHG treatment began nearly 2 years later. Orthodontic outcome was evaluated when CHG ended and at the end of follow-up using the Peer Assessment Rating (PAR) index and Little**’**s Irregularity Index (LII). Costs were calculated using medical records, estimated chairside times and average personnel and material costs.

**Results:**

The mean total costs including personnel and material costs during the follow-up were higher in the EG (€416) compared with the LG (€364) (*p* = 0.043). No statistically significant differences between the groups were found in the PAR and in the LII scores at the end of follow-up. The number of visits during CHG treatment was higher in the EG (13.3) compared with the LG (10.4) (*p* = 0.01).

**Conclusions:**

Earlier initiation of CHG treatment increased the personnel and material costs, number of appointments, and chairside time. Based on the PAR and in the LII scores, the orthodontic outcome of CHG treatment was equal regardless of the timing of the treatment. If orthodontic treatment during mixed-dentition stage is considered, it would be justified from an economic perspective to favor treatment during late mixed-dentition.

## Introduction

The optimal treatment timing of Class II malocclusion has been studied extensively. Opinions on the correct timing of treatment vary, and there are results supporting various approaches. Early treatment during the mixed-dentition stage has been stated to reduce skeletal jaw discrepancy [1–3] and prevent incisor trauma in Class II malocclusion [4, 5]. On the other hand, early therapy for Class II malocclusion has been stated to extend the total treatment time, increase the number of appointments, and incur additional costs [6]. Early treatment often leads to two-phased treatment and a longer total treatment time [7]. However, there are also findings suggesting that the timing of Class II malocclusion treatment has no effect on costs or the treatment outcome [8].

Orthodontic treatment causes costs for society, patients, and the patients’ guardians. Cost-effectiveness research in orthodontics has focused on comparing the outcomes and costs of different orthodontic appliances, aiming to find the most cost-effective method to achieve the desired outcome [9, 10]. Cost-minimization analyses (CMA) identify cost differences when the orthodontic outcome of treatment is the same [11]. In addition, cost-benefits can also be evaluated within orthodontic appliances based on their timing of use [6, 8]. While it is not reasonable to choose an orthodontic treatment method solely from an economic point of view, the costs of treatments must be considered as one aspect in treatment planning. Health economic research may help in designing orthodontic treatment plans and identifying effective treatment pathways.

In economic evaluations, relevant costs can be identified from different perspectives. From the perspective of healthcare, attention is focused on the costs incurred by the service provider and the cost-benefits of the treatment. From the patient’s perspective, the costs incurred by the patient or their guardians, such as service fees and absences from work, are emphasized. According to a recently published article, the societal perspective, which considers all costs to society, is the correct approach to a full economic evaluation [10]. The most common point of view in high-quality cost analyses of orthodontic treatment is the societal perspective (50%), the second most common is healthcare (38%), and the rest are various combinations of healthcare/patient and healthcare/societal perspectives [10]. Administrators, healthcare providers, patients, and the patients’ parents all view the costs of orthodontic treatment from their own perspectives. Although it may not be possible to determine or measure all costs, it is important to recognize them all [11].

In addition to cost comparison, it is important to reliably measure the outcome of the treatment. The assessment of orthodontic outcomes has generally been carried out using the subjective opinion and experience of clinicians [12]. Occlusal indices are used to minimize subjectivity when evaluating treatment needs and the outcomes of orthodontic treatment [13–15]. Even though validated instruments are used to assess occlusion, some variation is always present among professionals [13, 14]. The Peer Assessment Rating (PAR) is a validated method to evaluate orthodontic outcomes and the extent to which malocclusion deviates from normal occlusion and alignment [16–18]. The Little**’**s Irregularity Index (LII) has been demonstrated to be an appropriate measure for quantifying lower anterior crowding [19–21].

The primary aim of this study was to investigate the effect of cervical headgear (CHG) timing on treatment costs by evaluating the number and duration of appointments as well as personnel and material costs. The secondary aim was to assess the benefits of headgear timing on treatment outcomes by using occlusal indices.

## Material and methods

### Trial design and ethical approval

This CMA is a secondary analysis of the data from a previous randomized controlled trial [22–26]. The economic perspective of the study was added later, and therefore it has not been mentioned in the original registration.

The regional medical research ethics committee of the Wellbeing services county of North Ostrobothnia, Finland (EETTMK: 46/2003) and health center supervisors approved the trial set-up. The trial was conducted according to the Declaration of Helsinki.

### Sample size calculation

A pre-study power analysis for an RCT was calculated with GPower 3.1 Software using 80% power and a 0.05 significance level. The sample size calculation was based on a CHG trial with a similar study design using the means and standard deviations (SDs) of the ANB angle values [27]. Eleven participants per group were needed to reach sufficient power. To tolerate patient dropouts during the long-term follow-up, the number of participants was increased.

### Participants, inclusion criteria and exclusion criteria

Participants were recruited to this trial (from February 2004 to June 2008) from three public health centers in Northern Finland. The pool of subjects comprised 270 7-year-old children who were assessed for eligibility by orthodontists. The inclusion criteria for this trial were Angle Class II molar relationships, deep bite, and overjet over 6 mm. Patients with earlier orthodontic treatment, obstructive sleep apnea, continuous airway infections, inborn facial asymmetry, facial syndrome, or the maxillary-mandibular plane angle (MMPA) over 35 degrees were excluded from this trial. The need for orthodontic treatment was determined by a 10-grade scale based on the Treatment Priority Index (TPI) [28].

**Figure 1:**
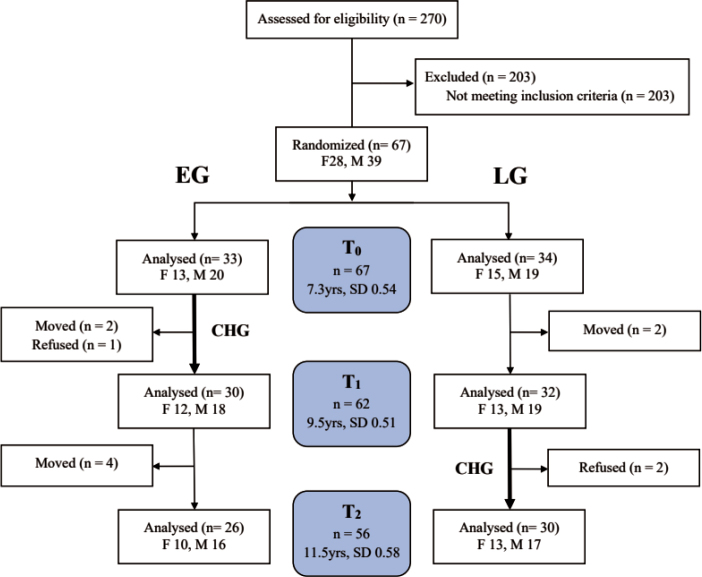
Flow chart of the trial. F: female; M: male; EG: early group; LG: later group; CHG: cervical headgear.

The flow of participants is presented in Figure 1. The total number of subjects fulfilling the inclusion criteria was 67 (early group [EG], *n* = 33; later group [LG], *n* = 34). The mean value of ANB was 5.2 (SD 1.92) in the EG and 5.4 (1.83) in the LG at T_0_ [22]. Based on cephalometric analysis, there were no skeletal differences between the groups at baseline [22]. No differences were observed between the groups in dental arch dimensions at baseline [25].

At the end of follow-up (T_2_), the total number of participants was 56 (EG, *n* = 26; LG, *n* = 30). The dropouts included eight children who had moved and three subjects who refused the treatment.

### Randomization and blinding

Oral and written information about the study protocol was given to all the children fulfilling the eligibility criteria and their guardians, and written consent was obtained before the study. The participants were randomized (1:1 ratio) into two equal-sized groups, the early and later groups. The randomization was made using sealed and opaque envelopes including numbered notes. The participants picked and opened one of the envelopes and gave it to the clinician to inform the treatment group. More specific details of the randomization process have been published in earlier papers concerning this trial [22–26].

Data analysts were blinded. All the analyzed material was coded so that the participants or groups could not be identified. Blinding of the personnel, patients, and the patients’ guardians could not be achieved during CHG treatment.

### Interventions

Appliance settings for the CHG treatment were identical among all participants. Activated force magnitude was 500 g. Children were instructed to wear CHG for 8–10 hours a day while sleeping. The inner bow of the CHG was expanded 5 mm, and the outer bow was bent 10 degrees upwards.

In the EG, the CHG treatment started after eruption of the first upper molars(7.8 yrs, interquartile range [IQR] 7.3–8.2). The EG was treated with CHG between time points T_0_ and T_1_. In the LG, the CHG therapy started nearly 2 years later compared with the EG after T_1_ (9.7 yrs, IQR 9.2–9.7). The LG was treated with CHG between time points T_1_ and T_2._ The treatment protocol was similar in both groups. The CHG treatment was carried out until Angle Class I molar relationships were achieved. Reduced use of CHG was applied when needed.

Orthodontic treatment was planned and supervised by an orthodontist who further delegated the procedures to general dentists and dental hygienists. During the orthodontic visits, general dentists and dental hygienists performed only orthodontic procedures.

### Outcomes

Main outcomes of the present study were total costs and the PAR score. Additional outcomes were LII, number of visits and chairside time during CHG treatment.

### Data handling

All scanning and measuring of dental casts were performed by one of the authors (M.H.). Appointment details for the cost analysis were collected from the electric patient records by the same author (M.H.).

### Cost measures

The approach chosen for the economic evaluation was to use the perspective of healthcare system. In this study, the focus was on personnel and material costs, number of appointments, and chair time. The material cost calculations were based on average commercial prices in 2022. The personnel costs in 2021 were collected from the Statistics Finland open database [29]. Treatment time was calculated by using the number of appointments and estimated average duration of an orthodontic appointment in each personnel category. The expenses of facilities, equipment, and maintenance were excluded from this analysis because they depend strongly on local factors and determining them accurately proved to be impossible. Indirect costs resulting from the guardian’s absence from work and loss of income when accompanying their child to the orthodontic appointments were excluded from this study due to the perspective chosen.

### Outcome measures

Dental casts were manufactured using alginate impressions and bite indexes, which were taken at T_1_ (9.5 years, SD 0.50) and at T_2_ (11.5 years, SD 0.62). Dental casts were scanned into digital 3D models (3Shape, R700TM Orthodontic Scanner, Denmark). The occlusion indices were measured from 3D models with an analyzer program (3Shape, OrthoAnalyzer^TM^ 2012, Denmark).

Evaluation of orthodontic outcome was based on the PAR index and LII. The PAR index consists of five components: anterior segments, buccal segments, overjet, overbite, and midline. These components are weighted and summed to the PAR total [16–18]. The occlusion is classified as an almost ideal (PAR < 5), acceptable (5 ≤ PAR ≤ 10), or unacceptable occlusion (PAR > 10) [16]. The LII describes mandibular anterior alignment [19, 20]. The lower anterior segment is determined into perfect alignment (LII = 0), minimal irregularity (1 ≤ LII ≤ 3), moderate irregularity (4 ≤ LII ≤ 6), severe irregularity (7 ≤ LII ≤ 9), and very severe irregularity (LII > 10).

Intra-examiner reliability of the PAR and LII measurements was determined by duplicating the measurements of 20 dental casts.

### Statistics

Differences in age at the beginning of CHG, total duration of CHG, PAR scores, LII scores, number of visits, costs, and chairside time between groups and genders were evaluated using the Mann-Whitney *U* test for continuous variables and the χ^2^ test for categorical variables. Some of the variables were non-normally distributed, and therefore Mann-Whitney *U* test was chosen, and both mean and median values as well as IQR were reported. The intraclass correlation coefficient (ICC) was evaluated using two-way analysis of variance (ANOVA). Analyses were carried out using IBM SPSS version 27 (SPSS. Inc., Chicago, IL, USA).

## Results

The treatment was carried out between time points T_0_–T_1_ in the EG and between T_1_ and T_2_ in the LG. The active phase lasted 1.6 years (SD 0.76) in the EG and 1.4 years (SD 0.80) in the LG.

### Intra-examiner reliability

The ICC value was 0.941 for PAR measurements and 0.989 for LII measurements. The ICC values indicated an excellent level of intra-examiner reliability.

### Costs

The personnel costs before CHG treatment were lower in the EG (€70) compared with the LG (€99) (*p* < 0.001; Table 2). In the EG, the personnel costs before CHG treatment were higher for females (€79) compared with males (€63) (*p* = 0.008; Table 1). The personnel costs during CHG treatment were higher in the EG (€259) compared with the LG (€193) (*p* = 0.004; Table 2). The total personnel costs during CHG treatment, including emergency and no-show visits, were higher in the EG (€303) compared with the LG (€228) (*p* = 0.004; Table 2).

**Table 1:** Description of the participants: mean and median values and IQRs for age at the beginning of CHG (years), total duration of CHG (months), costs (€), PAR score, LII, and number of visits according to group and gender.

Description of the participants	EG				LG			
	Female		Male		Female		Male	
	Mean	Md (IQR)	Mean	Md (IQR)	Mean	Md (IQR)	Mean	Md (IQR)
Age at the beginning of CHG (years)	7.9	8.0 (7.4–8.3)	7.7	7.7 (7.2–8.1)	9.4	9.4 (9.2–9.6)	9.9	9.6 (9.2–10.0)
Total duration of CHG (months)*	29.2	28 (16–38)	26.7	21 (18–35)	19.9	19 (12–28)	21.4	21 (13–29)
**COSTS (€)**								
Personnel cost before CHG	79^b^	78 (74–90)	64^b^	64 (46–73)	98	95 (75–108)	99	95 (74–120)
Personnel cost during CHG (scheduled)	262	234 (188–359)	257	244 (185–310)	187	148 (139–243)	199	185 (148–259)
Personnel cost, emergency during CHG	33	24 (15–46)	28	21 (9–44)	19	12 (0–24)	26	30 (6–37)
Personnel cost, No-show during CHG	13	0 (0–19)	15	0 (0–19)	11	0 (0–19)	13	0 (0–19)
**Total personnel cost during CHG**	308	314 (213–403)	300	302 (225–350)	216	173 (139–280)	238	217 (172–302)
**Total personnel cost (before + during)**	387	41 (281–489)	364	367 (289–418)	314	287 (235–369)	337	327 (272–388)
Material costs	36	30 (30–39)	38	39 (30–44)	35	32 (30–37)	33	30 (30–35)
Material cost, emergency	4	4 (0–6)	6	4 (0–9)	2	0 (0–2)	4	2 (0–4)
**Total material cost**	40	35 (32–45)	44	43 (34–52)	38	34 (30–42)	36	34 (30–37)
**TOTAL COSTS during CHG**	348	362 (244–454)	344	347 (255–402)	254	202 (174–313)	274	247 (202–338)
**TOTAL COSTS (during + before)**	427	457 (313–536)	408	414 (319–471)	352	320 (267–408)	373	366 (305–426)
**ORTHODONTIC OUTCOME**								
PAR when CHG ended**	18.2	17 (11–21)	18.5	17 (12–22)	16.0	15 (13–19)	18.6	16 (14–21)
PAR at T_2_	13.1	12 (9–18)	16.5	15 (12–21)	16.0	15 (13–19)	18.6	16 (14–21)
LII when CHG ended**	5.1^a^	5.2 (4.3–6.3)	3.4^a^	2.8 (2.0–3.7)	3.2	2.0 (1.4–4.7)	4.1	3.5 (1.8–6.9)
LII at T_2_	5.3	4.1 (2.9–6.8)	3.7	3.2 (1.7–5.8)	3.2	2.0 (1.4–4.7)	4.1	3.5 (1.8–6.9)
**VISITS**								
Scheduled visits before CHG	4.5	4 (4–5)	4.0	4 (3–4)	5.8	6 (4–7)	5.8)	6 (5–7)
Scheduled visits during CHG	13.8	13 (9–19)	10.1	12 (10–16)	10.1	10 (7–12)	10.6	9 (9–14)
Emergency visits related to CHG	2.2	2 (1–4)	1.8	2 (1–2)	1.2	1 (0–2)	0.7	2 (1–2)
No-show visits during CHG	0.7	0 (0–1)	0.8	0 (0–1)	0.5	0 (0–1)	1.5	0 (0–1)
**Total visits**	21.1	22 (15–26)	19.6	19 (15–23)	17.7	18 (14–21)	18.6	17 (16–22)

Gender differences evaluated using the Mann-Whitney *U* test and the χ^2^ test. CHG: cervical headgear; IQR: interquartile range; LII: Little**’**s Irregularity Index; PAR: Peer Assessment Rating; EG: early group; LG: later-timed group.

* Total duration of CHG treatment included active phase and reduced use phase.

** In the EG, CHG ended at T_1_. In the LG, CHG ended at T_2_.

a Statistically significant gender differences in EG: LII when CHG ended (*p* = 0.008).

b Statistically significant gender differences in EG: Personnel cost before (*p* = 0.008).

**Table 2:** Mean and median values and IQRs for age at the beginning of CHG (years), total duration of CHG (months), costs (€), PAR score, LII, and number of visits according to group, and significances of the differences between the groups.

Costs, outcome and visits	EG		LG		*p*
	Mean	Md (IQR)	Mean	Md (IQR)	
Age at the beginning of CHG (years)	7.8	7.8 (7.3–8.2)	9.7	9.4 (9.2–9.7)	< 0.001
Total duration of CHG (months)*	27.7	21 (18–35)	20.8	21 (13–28)	0.081
**COSTS (€)**					
Personnel cost before CHG	70	70 (58–84)	99	95 (76–120)	< 0.001
Personnel cost during CHG (scheduled)	259	241 (185–333)	194	174 (147–259)	0.004
Personnel cost, emergency during CHG	30	24 (12–43)	23	19 (0–37)	0.254
Personnel cost, no-show during CHG	14	0 (0–19)	12	0 (0–19)	0.987
**Total personnel cost during CHG**	304	314 (222–374)	229	212 (148–285)	0.004
**Total personnel cost (before + during)**	374	373 (293–470)	327	315 (250–371)	0.051
Material costs	37	37 (30–41)	34	32 (30–37)	0.100
Material cost, emergency	5	4 (0–8)	3	1 (0–4)	0.035
**Total material cost**	42	41 (34–51)	37	34 (30–38)	0.024
**TOTAL COSTS during CHG**	346	352 (255–422)	265	247 (183–319)	0.002
**TOTAL COSTS (before + during)**	416	520 (414–584)	364	350 (287–407)	0.043
**ORTHODONTIC OUTCOME**					
PAR when CHG ended**	18.4	17 (12–22)	17.5	16 (14–19)	0.655
PAR at T_2_	15.0	13 (10–17)	17.5	16 (14–19)	0.146
LII when CHG ended**	4.1	3.7 (2.7–5.6)	3.7	3.3 (1.6–5.8)	0.325
LII at T_2_	4.4	3.8 (2.3–6.6)	3.7	3.3 (1.6–5.8)	0.369
**VISITS**					
Scheduled visits before CHG	4.2	4 (3–5)	5.8	6 (5–7)	< 0.001
Scheduled visits during CHG	13.3	12 (10–18)	10.4	9 (8–12)	0.010
Emergency visits related to CHG	2.0	2 (1–3)	1.4	1 (0–2)	0.232
No-show visits during CHG	0.8	0 (0–1)	0.6	0 (0–1)	0.967
**Total visits**	20.2	19 (15–24)	18.2	18 (15–21)	0.193

Group differences evaluated using the Mann-Whitney *U* test. CHG: cervical headgear; IQR: interquartile range; LII: Little**’**s Irregularity Index; PAR: Peer Assessment Rating; EG: early group; LG: later-timed group.

* Total duration of CHG treatment included active phase and reduced use phase.

** In the EG, CHG ended at T_1_. In the LG, CHG ended at T_2_.

The mean total material costs of CHG treatment and emergency visits were slightly higher in the EG compared with the LG (*p* = 0.043, *p* = 0.035, respectively) although the absolute cost differences were small and not clinically meaningful (€42 vs. €36 and €5 vs. €3, respectively).

**Figure 2:**
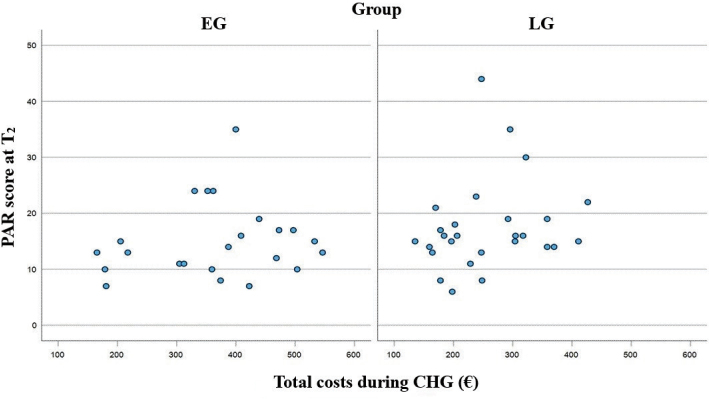
Dot-dash plot visualizing PAR scores at T_2_ and total costs during CHG (€) according to group and gender of participants. EG: early group, LG: later group; PAR: Peer Assessment Rating; CHG: cervical headgear.

The mean total costs including personnel and material costs during the follow-up were €416 for the EG and €363 for the LG (*p* = 0.043; Table 2). During the CHG treatment, the mean total costs including personnel and material costs were higher in the EG (€346) compared with the LG (€265) (*p* = 0.002; Tables 1 and 2; Figure 2).

### Orthodontic outcome

No statistically significant differences between groups or genders were found in the PAR scores at T_2_ (Tables 1 and 2; Figure 2). There were no significant differences in the LII scores between the groups at T_2_. In the EG, the LII scores were higher for females (5.1) compared with males (3.4) when CHG ended (*p* = 0.008; Table 1).

### Number of appointments

The mean number of visits before CHG treatment was lower in the EG (5.2) compared with the LG (5.8) (*p* < 0.001; Table 2). The number of visits during CHG treatment was higher in the EG (13.3) compared with the LG (10.4) (*p* = 0.01; Table 2). No statistically significant differences were found between groups in emergency and no-show visits.

### Chairside time

The mean total chairside time was 270 minutes for the EG and 207 minutes for the LG (*p* = 0.006; Table 3). Among all participants, the total chairside time for visits to general dentists was longer in the EG (215 minutes) compared with the LG (152 minutes) (*p* = 0.004; Table 3). The mean total chairside time among females was 278 minutes for the EG and 200 minutes for the LG (*p* = 0.019; Table 3). Among females, the total chairside time for appointments with general dentists was longer in the EG (216 minutes) compared with the LG (144 minutes) (*p* = 0.039; Table 3). No significant differences were found between the groups in the total chairside time for visits to orthodontists or dental hygienists.

**Table 3:** Mean and median values and IQRs for chairside time during CHG (min) including scheduled appointments and emergency appointments according to group and gender of participants, and dentistry personnel.

Chairside time during CHG (min)		EG		LG		*p*
		Mean	Md (IQR)	Mean	Md (IQR)	
**All**	Dental hygienist	52	45 (0–75)	47	45 (15–75)	0.924
	General dentist – Dental nurse	215	210 (165–270)	152	143 (105–214)	0.004
	Orthodontist – Dental nurse	3	0 (0–0)	8	0 (0–6)	0.320
	**Total**	270	240 (195–315)	207	185 (150–255)	0.006
**Female**	Dental hygienist	58	60 (8–90)	50	45 (15–75)	0.687
	General dentist – Dental nurse	216	225 (150–293)	144	120 (98–195)	0.039
	Orthodontist – Dental nurse	5	0 (0–5)	6	0 (0–3)	0.960
	**Total**	278	270 (198–368)	200	165 (128–250)	0.019
**Male**	Dental hygienist	48	38 (0–75)	45	30 (15–60)	0.832
	General dentist – Dental nurse	214	210 (161–259)	158	150 (105–225)	0.053
	Orthodontist – Dental nurse	2	0 (0–0)	9	0 (0–10)	0.303
	**Total**	263	233 (195–304)	212	195 (155–265)	0.110

Group differences evaluated using the Mann-Whitney *U* test. CHG: cervical headgear; IQR: interquartile range; EG: early group; LG: later-timed group.

### Harms

No harms were encountered.

## Discussion

### Main findings

The results of the present study show that the personnel costs, number of appointments, and chairside time during CHG treatment were higher in the EG compared with the LG. This study also shows that the timing of CHG treatment has no significant effect on the overall orthodontic outcome based on evaluation of indices.

### Interpretation

Earlier start of orthodontic treatment has been found to increase the number of visits when considering the total orthodontic treatment [30]. Present results showed that the earlier timing of CHG treatment increased the number of appointments. The same effect can be found with the twin-block appliance treatment [6]. By contrast, the timing of headgear activator treatment was found to have no effect on the number of visits [8]. One possible explanation for the increased number of visits in the EGs is the longer need for reduced use of orthodontic appliances during follow-up. Therefore, more appointments do not necessarily mean improved outcomes.

In addition to the increased number of visits, personnel costs were higher in the EG compared with the LG. Similar effects have been observed with the costs of earlier timing of twin-block appliance and overall earlier timing of orthodontic treatment [6, 30]. This is likely due to the typical challenge of early orthodontic treatment, where there is more growth remaining and treatment is started well before the growth spurt. Overall, the results revealed that total costs during CHG treatment were 30% higher in the EG. Cost differences may be small for individual patients, but when viewed at the population level, the differences can become significant. Therefore, orthodontists should have a general understanding of the costs associated with orthodontic treatments.

The results of the present study showed that the timing of CHG had no significant effect on the overall orthodontic outcome, which is consistent with earlier studies focused on twin-block appliance and headgear activator [6, 8]. Previous studies have demonstrated that the timing of headgear treatment has effects on individual dental measurements [22–26]. When examining individual measurements, the timing of orthodontic treatment may show differences between groups. However, when assessing the overall occlusion, occlusal indices are more comprehensive methods to describe orthodontic outcomes than individual measurements.

The PAR index is a widely-used method to assess orthodontic outcomes and the extent to which malocclusion deviates from normal alignment and occlusion [16–18]. The LII index has been found suitable for assessing lower anterior crowding, but it has also been criticized due to challenges with repeatability [19–21]. However, the PAR index has been used in previous CHG studies to describe the outcome of orthodontic treatment [7, 31], and the LII has been used to measure lower anterior alignment changes during CHG treatment [31].

Current results showed that the mean total chairside time during CHG treatment was longer in the EG compared with the LG. A previous study on the timing of headgear activator treatment found no differences in the chairside time between the groups [8]. The number of visits and the chairside time have a direct relationship with the costs: as the number of visits and chairside time increases, costs generally increase, and vice versa. Another finding in this study was that statistically significant differences in chairside time were observed between groups among females. Females in the LG had shorter chairside time compared to females in the EG, which could point to earlier dental development in females compared to males. The orthodontist’s chairside time was minimal, which can be attributed to the fact that most of the patients were not seen by the orthodontist during the headgear treatment. The orthodontic treatment was performed by the general dentists and dental hygienists, and the role of the orthodontists was to plan and supervise the treatment.

The average coverage of the orthodontic treatment among 0–18-year-olds is reported to be 11% in Finnish public healthcare, but the range between municipalities is wide, varying between 2 and 43% [32]. Class II malocclusion is the third most common reason for orthodontic treatment after anterior and posterior crossbite among children aged 7–9 years, and the most common reason among children aged 10–13 years within public healthcare services [33]. In Finland, the CHG is one of the most used appliances in the orthodontic treatment of children [32, 34].

Orthodontic treatment methods should not be chosen solely based on economic factors, but the costs of treatments must be considered due to limited resources. If earlier treatment timing is considered, it is reasonable to expect to have advantages over later treatment timing. When the outcomes are the same, it is reasonable to favor a shorter, more efficient, and lower-cost treatment approach. The burden of treatment on patients and parents cannot be ignored. This study provides important information on the impact of CHG treatment timing on the orthodontic outcome and costs, so that CHG could be used as efficiently as possible.

### Limitations, strengths, and generalization

The main limitation of this study is that it does not constitute a full economic evaluation. Thus, resource allocation cannot be based on this study. Another limitation is that the PAR and LII scores could not be measured at baseline, due to the early mixed-dentition phase. However, previous studies using the same dataset have not found differences in individual dental arch measurements between groups at baseline, supporting the validity of the outcome comparisons [22–26]. Limitation is also that the use of CHG was not monitored, so participants’ adherence to the treatment instructions cannot be confirmed. In this study, limitations are potentially caused by the fact that material was collected between 2004 and 2012, and in addition, the publication of the results has been delayed. The age of the material must be considered when interpreting the results.

The strength of this article is the use of an RCT design, and the outcome measures used have been shown to be valid and reliable [16–20].

Single-center design leads to limited generalizability and may cause potential bias. It is worth noting that the costs depend on local factors and cannot be extrapolated to other locations.

Interest in conducting an economic evaluation emerged after the initiation of the study. Economic evaluation was not mentioned in the study registration of the preceding RCT, which may have led to selective reporting bias.

This study represents a secondary analysis, and the sample size was not originally determined based on the present study outcomes. The sample size is too small to achieve adequate statistical power. Consequently, the analysis is at increased risk of Type II error, meaning that true effects may remain undetected, and this may have influenced the findings of the present study.

No correction for multiple comparisons was applied, but if all the null hypotheses are true, 5% of comparisons are expected to yield uncorrected *p*-values < 0.05 [35, 36]. With 78 statistical tests, this corresponds to about four chance significant results. In the present analyses, however, 16 *p*-values < 0.05 were observed. This number is greater than the ~4 expected by random variation alone, providing evidence that the observed effects are unlikely to be due to chance and instead support our study hypotheses.

## Conclusion

The current results showed that earlier timing of CHG treatment increased personnel and material costs, number of appointments, and chairside time. Regardless of the timing of CHG treatment, the overall orthodontic outcomes based on PAR and LII scores were equal. If orthodontic treatment during mixed-dentition stage is considered, it would be justified from an economic perspective to favor treatment during late mixed-dentition.

## Data Availability

The data underlying this paper will be shared by the corresponding author upon reasonable request.
